# The Be Our Ally Beat Smoking (BOABS) study, a randomised controlled trial of an intensive smoking cessation intervention in a remote aboriginal Australian health care setting

**DOI:** 10.1186/1471-2458-14-32

**Published:** 2014-01-13

**Authors:** Julia V Marley, David Atkinson, Tracey Kitaura, Carmel Nelson, Dennis Gray, Sue Metcalf, Graeme P Maguire

**Affiliations:** 1 The Rural Clinical School of Western Australia, The University of Western Australia, 12 Napier Terrace, PO Box 1377, Broome, WA 6725, Australia; 2 Kimberley Aboriginal Medical Services Council, 12 Napier Terrace, PO Box 1377, Broome, WA 6725, Australia; 3 Derby Aboriginal Health Service, 1 Stanley Street, PO Box 1155, Derby, WA 6728, Australia; 4 National Drug Institute, Curtin University, GPO Box U1987, Perth, WA 6845, Australia; 5 School of Medicine and Dentistry, James Cook University, Cairns, Queensland 4870, Australia; 6 Baker IDI, Alice Springs, Northern Territory 0871, Australia

**Keywords:** Indigenous, Aboriginal, Torres Strait Islander, Randomised controlled trial, Smoking cessation, Be Our Ally Beat Smoking (BOABS) Study

## Abstract

**Background:**

Australian Aboriginal and Torres Strait Islander peoples (Indigenous Australians) smoke at much higher rates than non-Indigenous people and smoking is an important contributor to increased disease, hospital admissions and deaths in Indigenous Australian populations. Smoking cessation programs in Australia have not had the same impact on Indigenous smokers as on non-Indigenous smokers. This paper describes the outcome of a study that aimed to test the efficacy of a locally-tailored, intensive, multidimensional smoking cessation program.

**Methods:**

A randomised controlled trial of Aboriginal researcher delivered tailored smoking cessation counselling during face-to-face visits, aiming for weekly for the first four weeks, monthly to six months and two monthly to 12 months. The control (“usual care”) group received routine care relating to smoking cessation at their local primary health care service. Data collection occurred at enrolment, six and 12 months. The primary outcome was self-reported smoking cessation with urinary cotinine confirmation at final follow-up (median 13 (interquartile range 12–15) months after enrolment).

**Results:**

Participants in the intervention (n = 55) and usual care (n = 108) groups were similar in baseline characteristics, except the intervention group was slightly older. At final follow-up the smoking cessation rate for participants assigned to the intervention group (n = 6; 11%), while not statistically significant, was double that of usual care (n = 5; 5%; p = 0.131). A meta-analysis of these findings and a similarly underpowered but comparable study of pregnant Indigenous Australian women showed that Indigenous Australian participants assigned to the intervention groups were 2.4 times (95% CI, 1.01-5.5) as likely to quit as participants assigned to usual care.

**Conclusions:**

Culturally appropriate, multi-dimensional Indigenous quit smoking programs can be successfully implemented in remote primary health care. Intensive one-on-one interventions with substantial involvement from Aboriginal and Torres Strait Islander workers are likely to be effective in these settings.

**Trial registration:**

Australian New Zealand Clinical Trials Registry (ACTRN12608000604303).

## Background

In 2008, the age-standardised prevalence of current smoking among Indigenous people was more than double that among other Australians (49.8% compared with 20.5% of those aged 18 years and over) [1]. Much of the health disparity between Indigenous and non-Indigenous Australians is attributable to conditions that are either due to or exacerbated by tobacco smoking. Ischaemic heart disease and type 2 diabetes contributes 14% and 12%, respectively, to the Indigenous health gap [2]. Indigenous men and women 35 – 54 years of age die from ischaemic heart disease at 7.2 and 16.6 times, respectively, the rate in the non-Indigenous Australian population [3]. In addition similarly aged Indigenous men and women die from chronic lung disease at a rate 9.7 and 13.9 times greater respectively, and from lung cancer at a rate twice that of non-Indigenous Australians [3]. Smoking substantially increases the risk of both macrovascular and microvascular complications of diabetes and may have a role in the development of type 2 diabetes [4]. Stopping smoking has been described as the single most important individual change Indigenous Australian smokers could make to improve their health.

While many smokers quit unassisted, for others helping them to become and remain non-smokers is difficult. Randomised controlled trials (RCTs) aimed at the general smoking population demonstrate interventions with success rate of at most 25% at 12 months and more typically substantially lower. In the general population smoking cessation interventions have an odds ratio (OR) of between 1.42 and 2.17, depending on the type of intervention used [5]. There have been several smoking cessation interventions conducted amongst disadvantaged and Indigenous groups [6,7]. However, there is a paucity of published RCTs [7].

Smoking cessation programs are a major priority in Indigenous Australian health. A range of strategies have been used to encourage Indigenous Australians to quit smoking however there have been few good quality studies that show which approaches work best. The only published Indigenous smoking cessation RCT investigating the benefit of a personal support intervention with validated smoking cessation showed a doubling of the smoking cessation rate after providing extra support (7% v 3%), although this improvement was not statistically significant [8].

While smoking cessation rates in primary health care settings are likely to be lower than in settings where patients have specifically sought help, particularly when health care providers approach patients to quit [8,9], more evidence of effective strategies are needed if effective policy is to be developed and implemented. Aboriginal Community Controlled Health Services (ACCHS) provide an appropriate setting for such interventions as they provide a culturally safe environment that has been shown to be successful in delivering interventions in other health care areas [10-12].

The Be Our Ally Beat Smoking (BOABS) Study aimed to test the efficacy of a culturally appropriate multidimensional intensive smoking cessation intervention provided by Aboriginal researchers in helping Aboriginal and Torres Strait Islander people to become and remain non-smokers at 12 months.

### Hypothesis

A culturally appropriate, multidimensional, intensive smoking cessation intervention, provided by trained Aboriginal researchers, will be more effective than current standard practice in achieving and sustaining cessation to tobacco consumption among Australian Aboriginal and Torres Strait Islander peoples.

## Methods

### Project staff

The project was undertaken at two ACCHS located in the remote towns of Derby (Derby Aboriginal Health Service – DAHS) and Kununurra (Ord Valley Aboriginal Health Service – OVAHS) in the Kimberley region of far north Western Australia [13] between January 2009 and June 2012. Aboriginal researchers were trained to deliver the program. These researchers came from various backgrounds: Aboriginal Health Worker (1), other Aboriginal health service employees (4) and people without a health background (4). For most of the study project managers were based at the centralised coordinating site (Kimberley Aboriginal Medical Services Council), in Broome. Investigators and research staff met before and at least annually during the trial to discuss all procedures. The Broome-based investigators (JM, DA, CN) met regularly to discuss any problems that arose during the trial.

### Study design and participants

The protocol for the BOABS Study has previously been described [13]. Briefly this study was a prospective, randomised, trial with parallel groups. Aboriginal and Torres Strait Islander smokers (current or who had quit within two weeks of enrolling) aged 16 years or older who were residents of Derby and Kununurra and who demonstrated any desire to quit smoking or cut down on the amount of cigarettes they smoked were recruited to the study. The exclusion criteria were: unable to provide informed consent; a health condition that would prevent them from completing the trial; or unlikely to be available for follow up at 12 months.

### Recruitment strategies

Participants were actively and opportunistically recruited. Active recruitment was facilitated by the Aboriginal researchers through incidental encounters in the community, family and community links. Participants were passively recruited through DAHS and OVAHS routine clinic visits as well as through running dedicated chronic disease prevention and screening clinics. Clients known or found to be current smokers were offered participation in the trial by health care providers. Recruitment originally intended to last 12 months eventually concluded at 30 months due to resource constraints (see below).

### Randomising and masking

As the planned intervention was labour intensive, participants were allocated to usual care or intervention groups in a 2:1 ratio. A computer generated random allocation sequence was used. Sealed envelopes containing the allocation were kept at the centralised coordinating site. Allocation occurred via telephone with envelopes being opened in sequential order for each site only when a participant had provided written consent and been enrolled in the study, eligibility confirmed, an identification number assigned, and the first questionnaire completed.

Due to the nature of the intervention the participants and research team were subsequently aware whether the participant belonged to the usual care or intervention group. Local healthcare staff may have been aware of this. The staff performing the urinary cotinine assay were blinded to the allocation.

### Procedures

Participants allocated to usual care received routine care relating to smoking cessation at their local primary health care service, including advice regarding quitting, pharmacotherapy, and self-initiated follow up. Participants allocated to the intervention group received usual care as above. In addition they were provided with smoking cessation counselling at face-to-face visits, which were scheduled weekly for the first four weeks, monthly to six months and two monthly to 12 months (12 sessions). The content delivered by Aboriginal researchers to participants in the intervention group included: motivational interviewing; diversions and strategies to deal with smoking triggers; action plans for preventing and dealing with short term relapses; discussion regarding the positives of smoking cessation; referral for and titration of pharmacotherapy; identification of factors driving smoking and case management to address these by linking participants with additional non-health support agencies (e.g. public housing, welfare, domestic violence and alcohol services); and strategies for smoking cessation-associated weight gain. They were also encouraged to attend a monthly smoking cessation peer support group. We planned that the Aboriginal researchers would organise weekly case conferences with the health care clinic medical officer/general practitioner to review all active participants in the intervention group.

### Study endpoints

Planned study endpoints were at six and 12 months following enrolment. The planned primary endpoint was not smoking at 12 months defined as self-reported non-smoking for at least seven days [8] and low urinary cotinine [14]. Final follow-up was extended to improve completeness, with 22 and 11 follow-ups from the usual care and intervention groups, respectively, occurring more than 15 months after enrolment. Multiple questions (Are you still a smoker? Have you smoked since the last BOABS Smoking Checkup? When was your last cigarette? On an average day how many cigarettes a day do you smoke?) were used to assess self-reported quitting. Urinary cotinine was analysed using tandem mass spectrometry (Waters 2795 Autosampler with Waters Quattro Premier Mass Spectrometer, Milford, MA USA) with a cut-off to indicate not smoking of < 50 ng/mL [15]. Secondary outcomes were the proportion of participants who: were not smoking at six months for at least seven days as determined by self-report; reported fair/poor health using a self-reported health questionnaire [16]; reported 20% or greater reduction in the number of cigarettes smoked each week; and various process evaluation indicators that assessed how well the intervention program was implemented according to the protocol (e.g. number of ‘face-to-face’ meetings completed). We also recorded quit attempts during the study.

### Sample size

The sample size estimation was based on a two-sided alpha of 0.05, power of 80%, ratio of intervention to control participants of 1:2, and an anticipated efficacy of maintaining smoking cessation at 12 months of 3% in the control group based on local program evaluation (personal communication CN) and 13% in the intervention group. Based on these assumptions the sample size required was 106 in the intervention group and 212 in the control group. To take into account those lost to follow up, the target sample size was 120 in the intervention group and 240 in the control group.

### Statistical analysis

Differences in baseline characteristics, and primary and secondary outcomes were compared using χ^2^ tests for categorical data, *t* tests for continuous normally distributed data and Mann–Whitney test for continuous non-parametric data. Changes from baseline to final follow-up of nicotine dependency score ≤5 (decreased dependency, no change, increased dependency) were compared using the Cochran-Maentel-Haenzel test for linear association. Fishers exact test and relative risks (RR) were used to compare smoking cessation endpoints between the intervention and control groups. Multivariate analyses did not significantly change the results and hence have not been included. All statistical tests were two-sided and a p value less than 0.05 was taken to be statistically significant.

### Systematic review and meta-analysis

Based on CONSORT recommendations of placing the results of this study into context [17] we carried out, post hoc, an associated systematic review of smoking cessation trials in Indigenous populations. Our selection criteria for inclusion in the meta-analysis were randomised controlled trials of interventions with validated smoking cessation in Indigenous populations. Our exclusion criteria were 1) trials comparing pharmaceutical interventions compared with placebo and 2) all studies where quitting smoking was not biochemically validated for all participants who self-reported they had quit smoking (e.g. urinary cotinine). A recent meta-analysis of four studies reported a RR of 1.43 (95% CI; 1.03-1.98) for smoking abstinence at 6–12 month follow-up in the intervention group, based on a very low quality of evidence [6]. Additional searches of the Cochrane Tobacco Addiction Group Specialised Register of Trials (June 2013), MEDLINE (June 2013), online clinical trial databases and publication references for potential studies were also conducted. The four trials reported in the recent review did not meet the selection criteria: two were not randomised [18,19], one assessed the efficacy of a pharmaceutical intervention (bupropion) compared with placebo [20], and one had smoking cessation validated in only a subset of participants who self-reported that they had quit smoking [21].

Only one published study [8] met these criteria and we combined our results with the data from this RCT. Although this study focused on pregnant women, like our study this was based on one-on-one intensive support compared with usual care. Both studies also allowed recent quitters to enrol and in line with other smoking cessation studies used 7-day point prevalence abstinence of smoking confirmed by cotinine to classify non-smokers [22]. An estimated pooled weight average for RRs was calculated using the Mantel-Hetzel fixed-effect model, with 95% confidence intervals. This meta-analysis was not part of the original study protocol.

### Ethics approval

The trial was conducted in compliance with the study protocol [13], the principles of Good Clinical Practice, the NHMRC National Statement on Ethical Conduct in Human Research (2007), NHMRC Australian Code for the Responsible Conduct of Research (2007), and NHMRC Values And Ethics: Guidelines For Ethical Conduct In Aboriginal And Torres Strait Islander Health Research (2003) [23-25]. The trial received approval from The University of Western Australia Human Research Ethics Committee and the Western Australian Aboriginal Health Information and Ethics Committee, and support from the Kimberley Aboriginal Health Planning Forum Kimberley Research Subcommittee.

## Results

**Figure 1:**
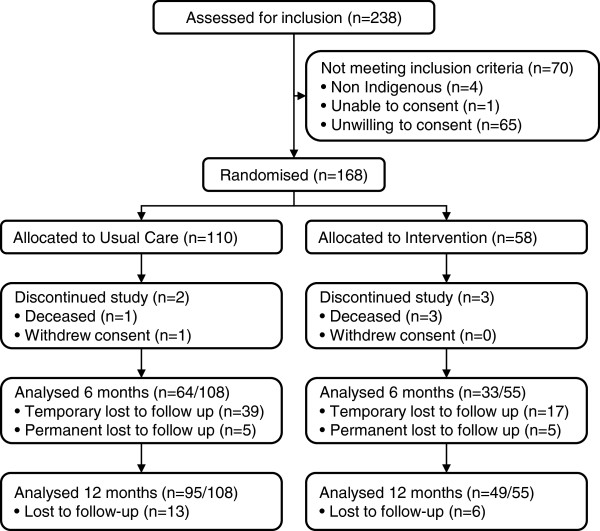
CONSORT flow of participants through study.

Participant recruitment occurred from January 2009 to May 2011 and follow-up occurred from February 2010 to July 2012. Common reasons that potential participants were unwilling to consent were a lack of willingness to be involved in a research project or not being interested in quitting smoking. Of the 168 participants who were randomised to the study, 163 continued in the study (Figure 1) and their baseline characteristics, and smoking and related risk behaviours were generally similar between the intervention and usual care groups (Tables 1 and 2). The small difference in mean age (38.3 v 41.9 years) between the intervention and usual care group was statistically but not clinically significant. Participants in both groups reported high levels of alcohol, cannabis and other illicit drug use (Table 2).

**Table 1:** Baseline characteristics of participants assigned to usual care and intervention groups

**Characteristic**	**Usual care group (n = 108)**	**Intervention group (n = 55)**
Age	38.3 (12.0)	41.9 (11.9)*
Female	54/108 (50%)	35/55 (64%)
Location		
Ord Valley Aboriginal Health Service	67/108 (62%)	35/55 (64%)
Derby Aboriginal Health Service	41/108 (38%)	20/55 (36%)
Socioeconomic		
Current employment	51/102 (50%)	30/53 (57%)
Formal education (year 10 or higher)	76/102 (74%)	35/51 (68%)
Long-term health conditions^†^	66/104 (63%)	35/52 (67%)
Self-reported health as fair/poor	30/106 (28%)	13/54 (24%)
Self-reported major event(s) that affected their smoking in the past 12 months^‡^	47/100 (47%)	20/51 (39%)

Data are number (%) or mean (standard deviation).

* *P* <0.05.

^†^Includes: high blood pressure; diabetes; kidney disease; eye problems not fixed by glasses; heart disease; chest/lung problems, including asthma, emphysema; ear or hearing problems; mental health problems; cancer.

^‡^Includes: death of family member or close friend, splitting up with partner, being in prison or a family member in prison.

**Table 2:** Baseline smoking and related risk behaviours of participants assigned to usual care and intervention groups

**Characteristic**	**Usual care group (n = 108)**	**Intervention group (n = 55)**
Smoking*		
Average number of cigarettes smoked per day	15 (10–25)	15 (11–25)
Years since started smoking	19.0 (10.9–26.7)	24.2 (15.0–31.6)
Age first started smoking	16 (14–18)	17 (15–19)
Nicotine dependency score ≤5 [26]	49/102 (48%)	24/53 (45%)
Self-reported desire to quit	63/105 (60%)	28/53 (53%)
Other drug use		
Current alcohol drinker	77/105 (73%)	41/53 (77%)
Smoked marijuana within the last month	37/105 (35%)	14/52 (27%)
Ever used other illicit drugs	17/104 (16%)	8/53 (15%)

Data are number (%) or median (interquartile range).

*Missing data for average number of cigarettes smoked per day: 4 usual care and 6 intervention; years since started smoking: 4 usual care and 2 intervention; age first started smoking: 4 usual care and 2 intervention.

An average of 7 of the 12 planned formal smoking cessation sessions were provided by the Aboriginal researchers to participants in the intervention group. Additional informal contact occurred throughout the study period (e.g. Aboriginal researchers meeting participants while out in the community and stopping to discuss how they are going with their quit smoking attempt). Twenty two (40%) participants in the intervention group had documented action plans. In addition to the content delivered by Aboriginal researchers during smoking cessation counselling sessions (see Methods), they also provided service mapping and linkages to other services. Individual case conferences with clinic staff did not happen as frequently as planned and only occurred as required. Few participants attended group sessions.

Clinic staff were reported to have provided the same level of routine care relating to smoking cessation to both groups: 23% of participants reported that they spoke to clinic staff about smoking during the study. Overall participants reported that clinic staff provided advice on risks of smoking (75%), recommended quitting smoking (71%), recommended medication to assist with quitting (54%), discussed passive smoking (61%), provided practical advice on quitting (50%), provided written advice on quitting (46%), arranged for a follow-up appointment (46%), and set a quit date (32%). There was no difference between groups. Participants were predominantly offered NRT Patches (39%) or varenicline (14%).

One hundred and forty-four (88%) participants were followed-up after 12 months, with 92 (56%) providing urine samples for validation of smoking cessation. Loss to follow-up was due to participants moving home and Aboriginal researchers not knowing where they moved to so they were unable to contact them.

For four participants there was a discrepancy between self-report smoking status and cotinine levels. Thirteen participants reported quitting smoking and 13 had a cotinine level consistent with not smoking. Two smokers (usual care group) claimed they had quit smoking at the final follow-up, however both reported quitting within the last 24 hours and had high cotinine levels. Two participants (intervention group) who self-reported continuing smoking had cotinine levels consistent with not smoking (below the level of detection of 20 ng/mL). One of these participants had the urine sample collected three months after the final questionnaire was administered and reported not smoking at the time of urine collection, no reason was identified for the other discrepancy as the participant was clear she was still smoking. As participants were only classified as not smoking if they satisfied both criteria, these four participants were classified as still smoking at final follow-up.

Participants in the intervention group quit smoking sooner than those in the usual care group: all intervention group participants reported quitting at least two months before the final follow-up, as against only three of five in the usual care group. There was no difference in the median time between enrolling and final follow-up for both groups (13 (IQR 12–15) months).

**Figure 2:**
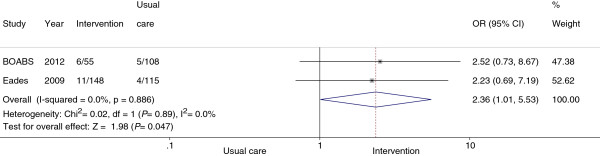
Comparison of smoking cessation for ‘usual care’ versus ‘intervention’ of randomised controlled trials involving Indigenous people*. * Smoking cessation was verified by cotinine, assuming those lost to follow-up were still smoking.

The proportions quitting smoking at final follow-up are shown in Table 3. Based on cotinine results alone at final follow-up the intervention group had a significantly higher proportion who quit smoking than the usual care group. However, based on the combination of self-report and cotinine level at final follow-up, the final results (2.4; 95% CI, 0.8-7.4, p = 0.131) were not statistically significant. A meta-analysis of the BOABS Study and the only other published RCT of a personal support intervention with validated smoking cessation [8] demonstrated a statistically significant higher smoking cessation rate for participants in intervention compared to usual care groups (Figure 2).

**Table 3:** Number of BOABS Study participants who had stopped smoking at final follow-up

**Outcome**	**Usual care group**	**Intervention group**	P*	**RR (95% CI)**^**†**^
Urinary cotinine <50 ng/mL	5/64 (8%)	8/28 (29%)	0.009	3.7 (1.3–10.2)
Self-report quitting and urinary cotinine <50 ng/mL for participants with complete outcome data	5/95 (5%)	6/49 (12%)	0.135	2.3 (0.7–7.2)
Self-report quitting and urinary cotinine <50 ng/mL assuming those lost to follow-up were still smoking	5/108 (5%)	6/55 (11%)	0.131	2.4 (0.8–7.4)

Data are number (%) based on the study arm to which participants were originally assigned;

*Fishers exact;

^†^Compared with usual care group;

No-one who had been recently incarcerated, chewed tobacco, or drank alcohol daily quit smoking. Age and gender were not independently associated with quitting. There was no relationship between the easily quantifiable measures of the intervention (documented action plan, or plan to stop smoking, setting a quit date and number of smoking cessation counselling sessions) and success in stopping smoking at final follow-up. We could not measure the quality of the relationships between the researchers and participants, which was likely to be important.

Secondary endpoints are summarised in Table 4. Follow-up at six months was only 59% (Figure 1) with seven of the 64 (11%) and three of the 33 (9%) participants in the usual care and intervention groups, respectively, self-reporting quitting smoking at six months. This was not validated with urinary cotinine.

**Table 4:** Secondary endpoints of BOABS Study participants

	**Usual care group**	**Intervention group**
Self-reported health as fair/poor	21/94 (22%)	14/49 (29%)
Nicotine dependency score ≤5 [26]^†^		
Baseline	26/50 (52%)	11/27 (41%)
Final follow-up	34/50 (68%)	18/27 (67%)
Reduced average daily cigarettes by ≥20%	38/74 (51%)	15/35 (43%)
Attempted to quit at any time during the study	36/90 (40%)	27/48 (56%)

Data are number (%) based on the study arm to which participants were originally assigned;

^†^Only included participants who had a nicotine dependency score recorded at baseline and final follow-up.

## Discussion

The current study did not demonstrate a statistically significant benefit from the BOABS intervention and due to difficulties recruiting participants did not have the power to do so. Pooling our data in a meta-analysis of the only other reported Indigenous smoking cessation RCT using a personal support intervention and validated smoking cessation, demonstrated a statistically significant increase in quitting (OR 2.4, 95% CI 1.01-5.5). This is similar to smoking cessation interventions in non-Indigenous settings (OR 1.4-2.2) [5]. Doubling the quit rate is a clinically important improvement and based on these pooled data one-on-one intensive interventions delivered by, and provided to, Indigenous Australians through a primary healthcare setting are more effective than usual care in encouraging people to stop smoking.

Both studies while more intensive than usual care were less intensive than originally planned. The participants in the BOABS intervention group received an average of seven of 12 smoking cessation sessions over 12 months and 40% had documented action plans for helping them to quit smoking. In contrast only 23% of participants in the usual care group reported discussing smoking cessation with clinic staff. In the non-Indigenous setting a systematic review of physician advice found increasing the intensity of the intervention showed a small advantage over minimal advice (RR 1.37; 95% CI 1.2-1.6) [27]. The level of intensity in these studies varied from brief intervention to repeated advice to quit as an inpatient with follow up in a special clinic.

Increasing the intensity of the intervention may have had an impact on the quit rate in the intervention groups of both the Eades study and BOABS. Although the Australian Indigenous smoking prevalence is high, there was only a 3% quit rate in a local program delivered at one of the sites prior to the BOABS intervention (personal communication CN). The quality of the relationships between the BOABS Aboriginal researchers and participants may well have had an impact. A qualitative evaluation of the observations and notes from the BOABS Study is required to further elucidate this.

There was repeated clinic and research staff training about roles and responsibilities to minimise contamination between groups. The process of regular follow up, the core of the intervention, appeared to be relatively free from contamination. Nonetheless even if some contamination did occur it was likely to reduce the difference between the intervention and usual care groups.

A limitation of both this study and the study by Eades and others was the lower than expected numbers of participants and an attendant lack of statistical power. While the BOABS Study had a high follow-up rate for a relatively long-term community-based RCT (88% actual follow-up v 90% planned), we were unable to recruit the planned number of participants. In this study there were a number of reasons why people chose not to enrol even though they wanted assistance to help quit smoking and most of these related to the consent and/or the randomisation processes. Therefore, we believe that more people will be interested in joining a program than a research study. It is unclear whether the people who did not want to participate in research would be more or less likely to quit than those who did participate.

Despite more than doubling the time for recruitment only about half of the expected number of participants were recruited. While we have demonstrated it is possible to undertake high quality RCTs in Aboriginal primary health care settings [28] the difficulty of enrolling participants in future studies should not be underestimated and needs to be carefully considered in planning.

Solutions to the problems of insufficient numbers and contamination include using more sites in multicentre trials and randomising at the cluster level. Individually randomised multicentre drug trials have been demonstrated to be possible in Aboriginal and Torres Strait Islander health [28]. Cluster randomised multicentre trials may be another solution for more complex non-placebo controllable interventions such as BOABS, however the risks of: 1) heterogeneity between sites; 2) the intervention only working in some locations; 3) added complexity of both design and analysis; 4) a substantially larger sample size required to obtain the same statistical power; 5) post-randomisation selection bias if randomisation occurs prior to participant recruitment; and 6) a potential slowdown of recruitment in the clusters that are to receive the less interesting treatment such as “usual care” [29-31], remain.

In addition to the risks listed above, RCTs are not well placed to take into account the need for operational changes of health service providers to deliver an intervention such as BOABS [30]. In the BOABS Study, because it was an RCT and by definition could not be delivered as part of usual health care, the intervention was delivered by individual workers and by design was not intended to create substantial change to the actions of other health care staff or the day to day operation of the clinics. We believe this had a negative effect on both recruitment and the intervention.

Although RCTs are generally considered the gold standard in research, it has been argued that in complex public health interventions other methodologies should be pursued [30]. Similarly RCTs may not be the best design for complex clinical individualised interventions such as BOABS. Alternative methodologies include plausibility (observational design with a comparison group) and adequacy (process indicators and outcome data are used to suggest if the intervention is having an important effect) evaluations [30]. While these methodologies provide lower levels of evidence, recruitment would be expected to be higher as it is not restricted in the same way it is in RCTs, and they should have greater potential to be generalisable. Future and ongoing evaluation of smoking cessation programs in this setting should therefore consider other methodologies beyond that of RCTs.

## Conclusion

Whilst the BOABS study did not demonstrate a significant benefit from a multi-dimensional smoking cessation intervention the meta-analysis of our and Eades’ earlier study does demonstrate that one-on-one intensive intervention delivered by and provided to Aboriginal and Torres Strait Islander peoples in a primary healthcare setting is more effective than usual care in encouraging smoking cessation. Taking into account the feasibility and cost-effectiveness of RCTs and cluster randomised trials, the priority should be to demonstrate the effectiveness of programs based on personal support to quit smoking in a real world setting. The questions should be whether real world interventions work in practice, whether they are cost effective and sustainable and to identify enablers and barriers to the integration of such programs into primary health care for Aboriginal and Torres Strait Islander peoples.

## Abbreviations

ACCHS: Aboriginal community controlled health service; BOABS: Be Our Ally, Beat Smoking; DAHS: Derby Aboriginal Health Service; DSMC: Data safety and monitoring committee; HREC: Human research ethics committee; NRT: Nicotine replacement therapy, available in a range of forms such as patch, gum lozenges, tablets and nasal spray; NHMRC: National Health and Medical Research Council; IQR: Interquartile range; OR: Odds ratio; OVAHS: Ord Valley Aboriginal Health Service; RCT: Randomised controlled trials.

## Competing interests

The authors declare that they have no competing interests.

## Authors’ contributions

All of the authors made contributions to the design of the ‘BOABS Study’. JM is an investigator and managed the project from September 2010 until it was completed. She designed the quantitative database and contributed to statistical analysis, and drafted the manuscript. TK is an Aboriginal researcher. She was the main researcher at DAHS and recruited participants; delivered the intervention in Derby and the 12-month follow-up in Derby and Kununurra. JM and TK were responsible for data collection. JM, DA and CN provided regular support to Aboriginal researchers delivering the intervention. GM led the statistical analysis of the quantitative data. All authors contributed to the interpretation of the findings and provided critical review of the manuscript. All authors read and approved the final manuscript.

## Pre-publication history

The pre-publication history for this paper can be accessed here:

http://www.biomedcentral.com/1471-2458/14/32/prepub
